# Antigenicity of Bovine Pericardium Determined by a Novel Immunoproteomic Approach

**DOI:** 10.1038/s41598-017-02719-8

**Published:** 2017-05-26

**Authors:** Katherine V. Gates, Ailsa J. Dalgliesh, Leigh G. Griffiths

**Affiliations:** 1 Department of Veterinary Medicine: Medicine and Epidemiology, University of California, Davis, One Shields Avenue, Davis, CA 95616 USA; 2 Department of Cardiovascular Diseases, Mayo Clinic, 200 First Street SW, Rochester, MN 55905 USA

## Abstract

Despite bovine pericardium (BP) being the primary biomaterial used in heart valve bioprostheses, recipient graft-specific immune responses remain a significant cause of graft failure. Consequently, tissue antigenicity remains the principal barrier for expanding use of such biomaterials in clinical practice. We hypothesize that our understanding of BP antigenicity can be improved by application of a combined affinity chromatography shotgun immunoproteomic approach to identify antigens that have previously been overlooked. Liquid Chromatography Tandem Mass Spectrometry (LC-MS/MS) analysis of affinity chromatography purified antigens resulted in identification of 133 antigens. Most importantly, antigens were identified from all subcellular locations, including 18 integral membrane protein antigens. Critically, isoforms of several protein families were found to be antigenic suggesting the possibility that shared epitope domains may exist. Furthermore, proteins associated with immune, coagulation, and inflammatory pathways were over-represented, suggesting that these biological processes play a key role in antigenicity. This study brings to light important determinants of antigenicity in a clinically relevant xenogeneic biomaterial (i.e. BP) and further validates a rapid, high-throughput method for immunoproteomic antigen identification.

## Introduction

Biomaterial antigenicity represents the primary barrier to expanding the use of xenogeneic biomaterials in clinical practice^1^. It has been shown that these responses are controlled by antibodies generated against both galactose-α-1,3-galactose (gal) and non-gal antibodies^2^. Although fixed and unfixed xenogeneic biomaterials are commonly used in clinical practice, recipient graft-specific immune responses limit their long term durability and functional outcomes^3, 4^. Clinical results achieved with such biomaterials are far from ideal, with both acute rejection (i.e., decellularized)^5^ and chronic rejection (i.e., glutaraldehyde-fixation) responses being reported^6^. Bovine pericardium (BP) has a native extracellular matrix (ECM) architecture that encompasses apposite structure/function properties and an essential ECM niche environment ideal for cell migration and proliferation^7^. However, ameliorating xenogeneic tissue antigenicity prior to implantation is critical in avoiding recipient graft-specific *in vivo* immune responses^8^. In an attempt to produce immunologically-acceptable xenogeneic tissue ECM scaffolds for clinical application, decellularization approaches have largely focused on ECM scaffold cellularity as the primary predictor of recipient graft-specific response^7, 9^. However, scaffold cellularity is a poor predictor of *in vivo* recipient graft-specific immune response and insufficient removal of antigens in decellularized tissue has potential to result in a catastrophic graft-specific immune response^10^. Recent studies have demonstrated that specific assessment and removal of ECM scaffold antigens prior to *in vivo* implantation more strongly correlates with reduced recipient graft-specific adaptive immune responses^11^. Future progress in the field of xenogeneic tissue scaffolds is therefore reliant on increased understanding of the antigenicity of such biomaterials, driving the need for high-throughput methods of antigen identification. In identifying the determinants of xenoantigenicity, we can improve our understanding and monitoring of immune responses toward clinically utilized biomaterials and inform efforts designed to reduce antigenicity of next generation xenogeneic biomaterials.

Immunoproteomic identification of antigenic determinants of xenogeneic biomaterials has previously relied on the use of two-dimensional (2-DE) Western blots, which are time consuming, and suffer from challenging reproducibility and limited capacity for separation of highly lipophilic proteins^12, 13^. The process of creating large-format 2-DE gels, running associated Western blots, isolating antigenic protein spots, and submitting samples for further proteomic analysis can take weeks for each positive protein identification^14^. Additionally, the commonly utilized practice of in-silico comparison between Western blots and duplicate gels introduces a potential source of error in ultimate identifications, particularly when considering the challenging reproducibility of 2-DE based approaches^15^. Furthermore, the limited dynamic range, particularly of silver stained gels, further reduces the accuracy of antigen quantification^15^. Finally, the challenges inherent in maintaining protein solubility during isoelectric focusing (IEF) limit the ability of 2-DE approaches to resolve highly lipophilic membrane proteins^13^. Despite the limitations of 2-DE Western blot approaches, longitudinal increase in graft-specific humoral responses has been demonstrated, with antigens identified from a variety of tissue and subcellular locations, although such progress remains challenging^14^. On the other hand, shotgun proteomic approaches have gained popularity due to their simplified workflow, enhanced reproducibility and compatibility with a broad range of protein solubilities^16^. Consequently, an affinity chromatography immunoproteomic approach for antigen identification has potential to overcome many of the limitations inherent with 2-DE methods.

We hypothesize that an affinity chromatography immunoproteomic approach can identify BP antigens and improve understanding of BP antigenicity. This approach has potential to overcome the limitations of 2-DE approaches and elucidates critical characteristics of non-gal xenoantigens. This study determines: (1) ability of an antibody affinity chromatography column to differentially capture antigenic proteins using a rabbit model of xenogeneic tissue implantation, (2) the spectrum of antigenic protein subcellular localizations available for identification using an affinity chromatography immunoproteomic antigen identification approach, and (3) the predominant subcellular locations and biologic processes in which antigens are located in a clinically applicable heart valve biomaterial (i.e., BP).

## Results

### IgG binding and cross-linking efficiency

**Figure 1:**
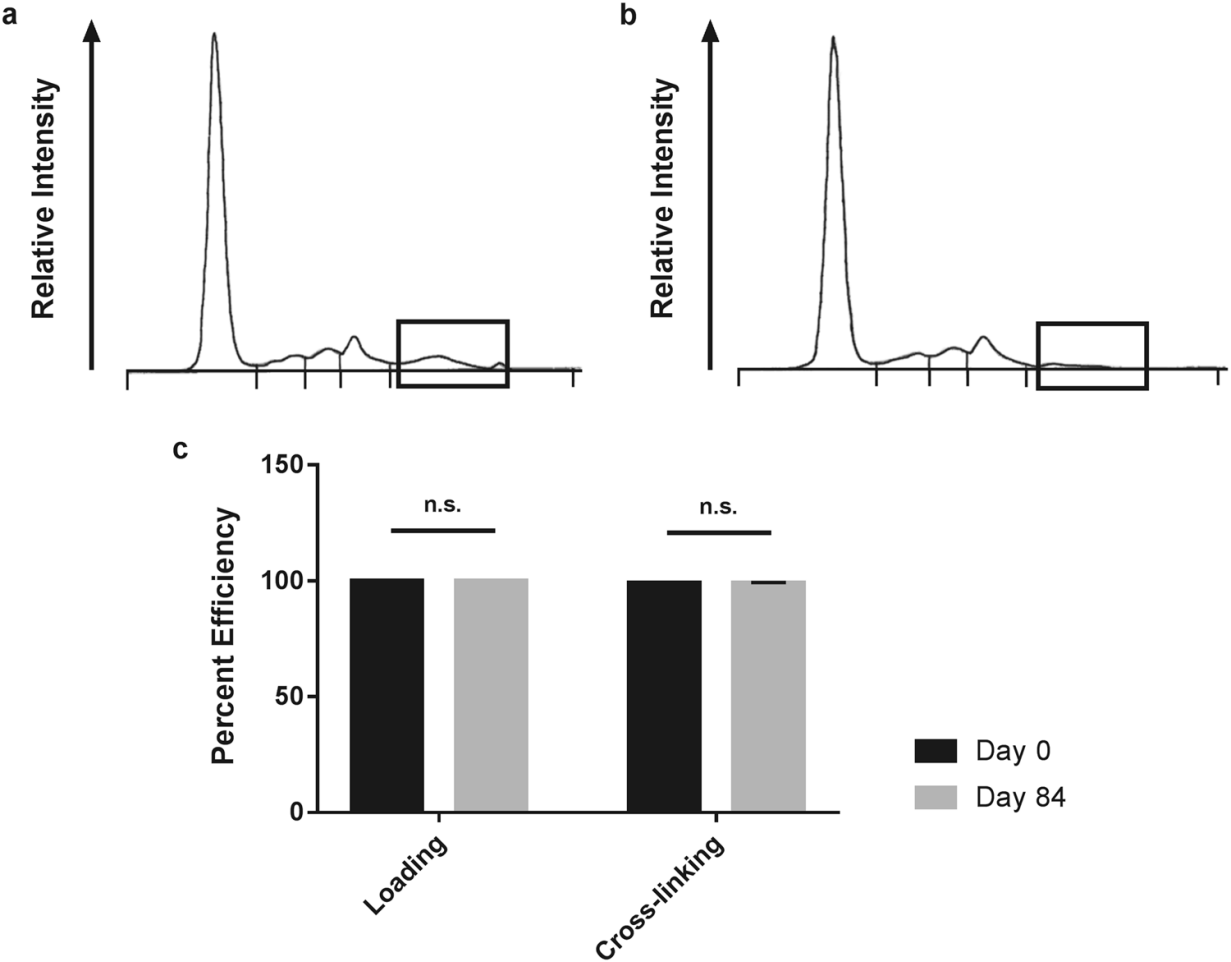
Assessment of column IgG binding capacity and cross-linking efficiency. Serum protein electrophoresis (SPE) of native rabbit serum showing IgG levels (**a**). Column run-through following D84 anti-native BP rabbit serum loading on SpinTrap Column (**b**). Boxes indicate the gamma peak, containing IgG along with fibronectin and C-reactive protein. Gamma peak was reduced from 0.3 mg/dL in native serum to below 0.1 mg/dL in the run-through (*n* = 1). Calculated efficiency of rabbit IgG capture and cross-linking to the column determined by ELISA analysis of IgG content in native serum and column run-through (**c**). No difference in IgG capture or cross-linking efficiency was found between D0 versus D84 serum using a paired two-tailed Student’s t-test (*n* = 6 per timepoint, *p* = 0.4663, values represent the mean ± s.d.).

Efficiency of IgG binding and cross-linking to SpinTrap columns was confirmed by performing serum protein electrophoresis (SPE) and IgG ELISA. SPE demonstrated reduction in gamma peak of run-through serum, while all other SPE peaks were unchanged (Fig. 1a and b). To further confirm and quantify column IgG binding capacity and cross-linking efficiency, rabbit IgG ELISA was run on serum, column run-through, and all subsequent wash steps from rabbits exposed to native BP at day 0 (D0) and day 84 (D84) post-immunization. Both D0 and D84 columns demonstrated a binding capacity of 99.99% (99.99 ± 0.0004% vs 99.99 ± 0.0005% respectively) of the IgG in native serum. By subtracting the IgG lost during subsequent washes from the amount initially bound to the column, cross-linking efficiency was calculated at 98.94 ± 0.26% for D0 and 99.08% ± 0.26% for D84 columns. Cross-linking and loading for D0 and D84 were not significantly different (*n* = 6, *p* = 0.4663) (Fig. 1c).

### Validation of Specificity of Antigenic Protein Capture in the Affinity Column

Western blotting was used for initial validation and visualization of specificity of antigen capture and elution from the affinity column. 1% (w/v) sodium dodecyl sulfate lipophilic (**SDS-L**) extracts resulted in Western blots with no apparent antigen capture. Consequently, only 134 mM non-detergent sulfobetaine-256 hydrophilic (**NDSB-H**), 1% (w/v) n-dodecyl-β-D-maltoside lipophilic (**NDSB-L**) and 0.1% (w/v) sodium dodecyl sulfate hydrophilic (**SDS-H**) extracts were utilized for later LC-MS/MS identification experiments. Percent protein yield was significantly lower in **NDSB-L** extracts (0.0655 ± 0.015%) than either **NDSB-H** (0.3152 ± 0.046%) or **SDS-H** (0.1735 ± 0.033%) (*p* < 0.05) (Supplemental Fig. 1).

**Figure 2:**
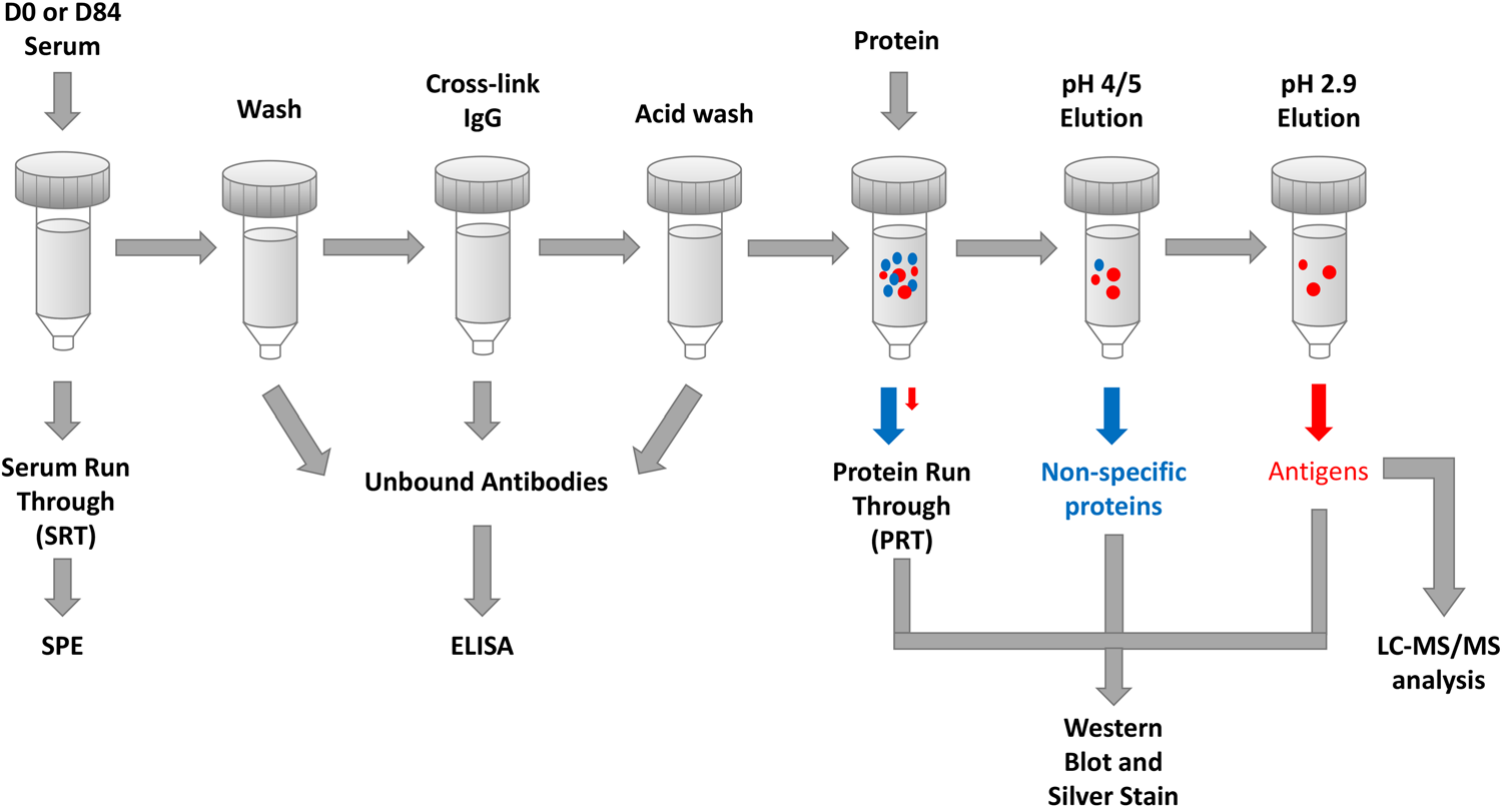
Diagram of workflow for affinity chromatography column generation and validation for antigen identification. Column IgG capture following serum loading was assessed using serum protein electrophoresis (SPE). Capture and cross-linking efficiency of IgG antibodies was determined using rabbit IgG specific ELISA. Specificity of antigen capture and non-specific protein binding were assessed using silver stained gels and Western blots with post-immunization rabbit serum (D84), for protein run-through from columns eluted at pH 5, 4 and 2.9. Antigen identifications were made using LC-MS/MS analysis of specifically bound proteins eluted from D0 versus D84 columns.

**Figure 3:**
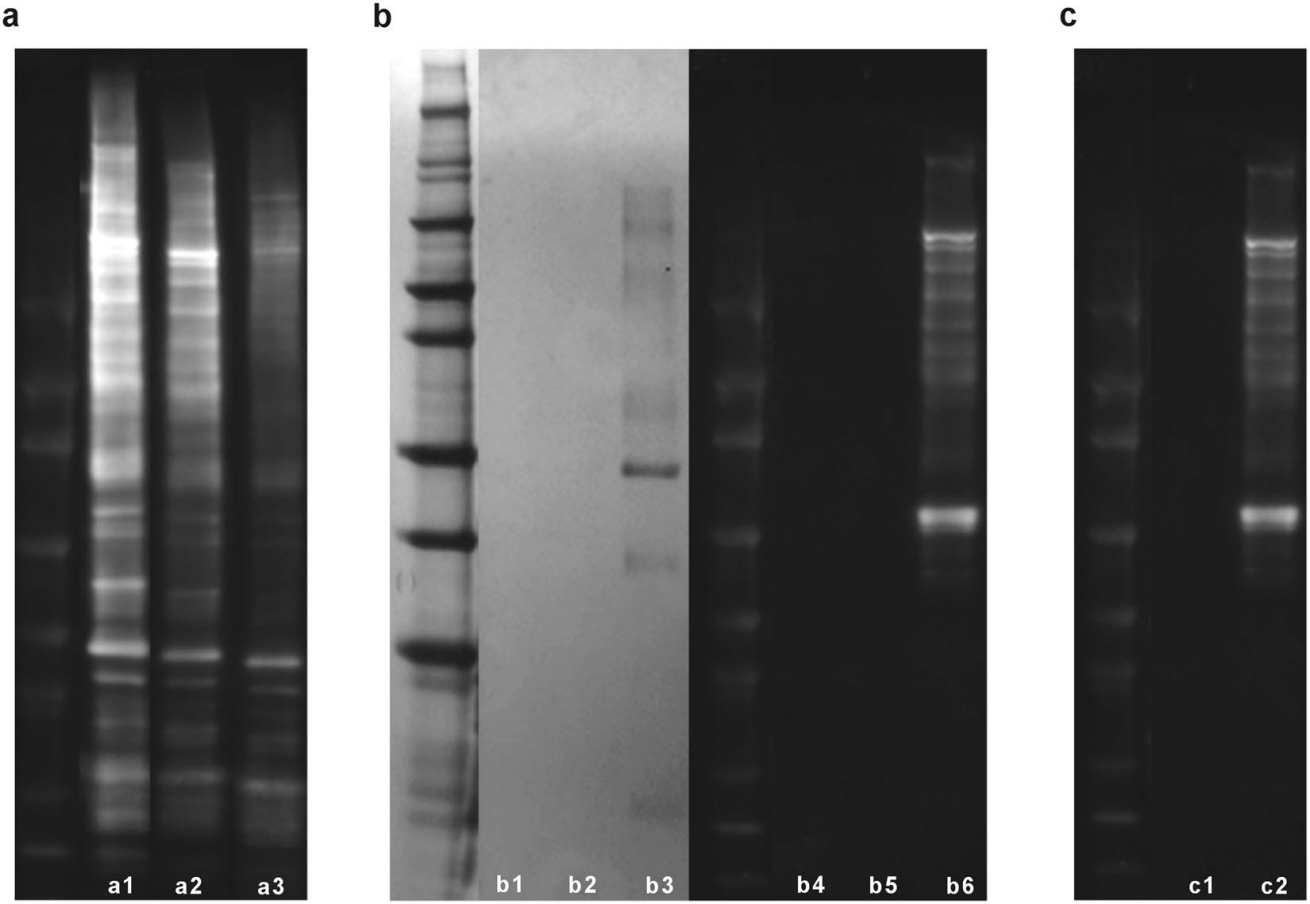
Western blot and silver stain of protein eluates. Western blot of total native protein extract (a1), protein extract run-through from D0 column (a2), and protein extract run-through from D84 column (a3). Both D0 and D84 columns demonstrate decreased antigenic protein content in column run-through, with greater removal of antigenic bands in D84 column. Silver stain for SDS-page gel of D84 pH 5, 4, and 2.9 eluates ((b1,b2 and b3) respectively) and corresponding D84 Western blot from pH 5, 4, and 2.9 eluates ((b4,b5 and b6) respectively), confirming that minimal non-specific binding is present and that all specifically bound proteins (pH 2.9 eluates) are antigenic. Comparison of D0 and D84 eluates at pH 2.9, demonstrating that more antigens are specifically captured from D84 columns (c2) than from D0 columns (c1). (*n* = 4 per extraction method and timepoint). Western blot and silver stained gel were cropped for easier visualization, uncropped images are available in the supplemental material.

**NDSB-H**, **NSDB-L** and **SDS-H** were incubated in D0 and D84 SpinTrap columns and subsequent protein run-through and eluates were collected (Fig. 2). Protein run-through from D0 columns demonstrated minimal reduction in antigenic band intensity compared to native BP protein extract (Fig. 3a), indicating that antigen capture in the column was minimal. There was a greater capture of antigenic proteins in D84 columns compared to D0 columns, as demonstrated by the greater reduction in band intensity of protein run-through from D84 columns (Fig. 3a and Supplemental Fig. 2a).

To determine if captured proteins were specifically bound to both D0 and D84 columns, stepwise pH elution was undertaken. Silver staining of eluates from both columns demonstrated minimal protein elution for pH 5 and 4 eluates, with elution of specifically captured proteins in pH 2.9 eluates (Fig. 3b and Supplemental Fig. 2b). Western blot analysis demonstrated that no antigenic proteins eluted off either column with washes at pH 5 and 4, but when the column was washed at pH 2.9 bound antigens were eluted (Fig. 3b and Supplemental Fig. 2a).

Finally, antigenic proteins eluted from D0 versus D84 were compared to determine if antigen capture was increased in columns generated using post-immunization serum compared to pre-immunization serum. D84 pH 2.9 eluate contained many more antigenic protein bands than the D0 pH 2.9 eluate, which identified virtually no antigenic protein bands (Fig. 3c and Supplemental Fig. 2a). This process was repeated for all four native protein extract types to confirm specificity of antigen capture for each extraction.

### Antigen Identification Efficiency

**Figure 4:**
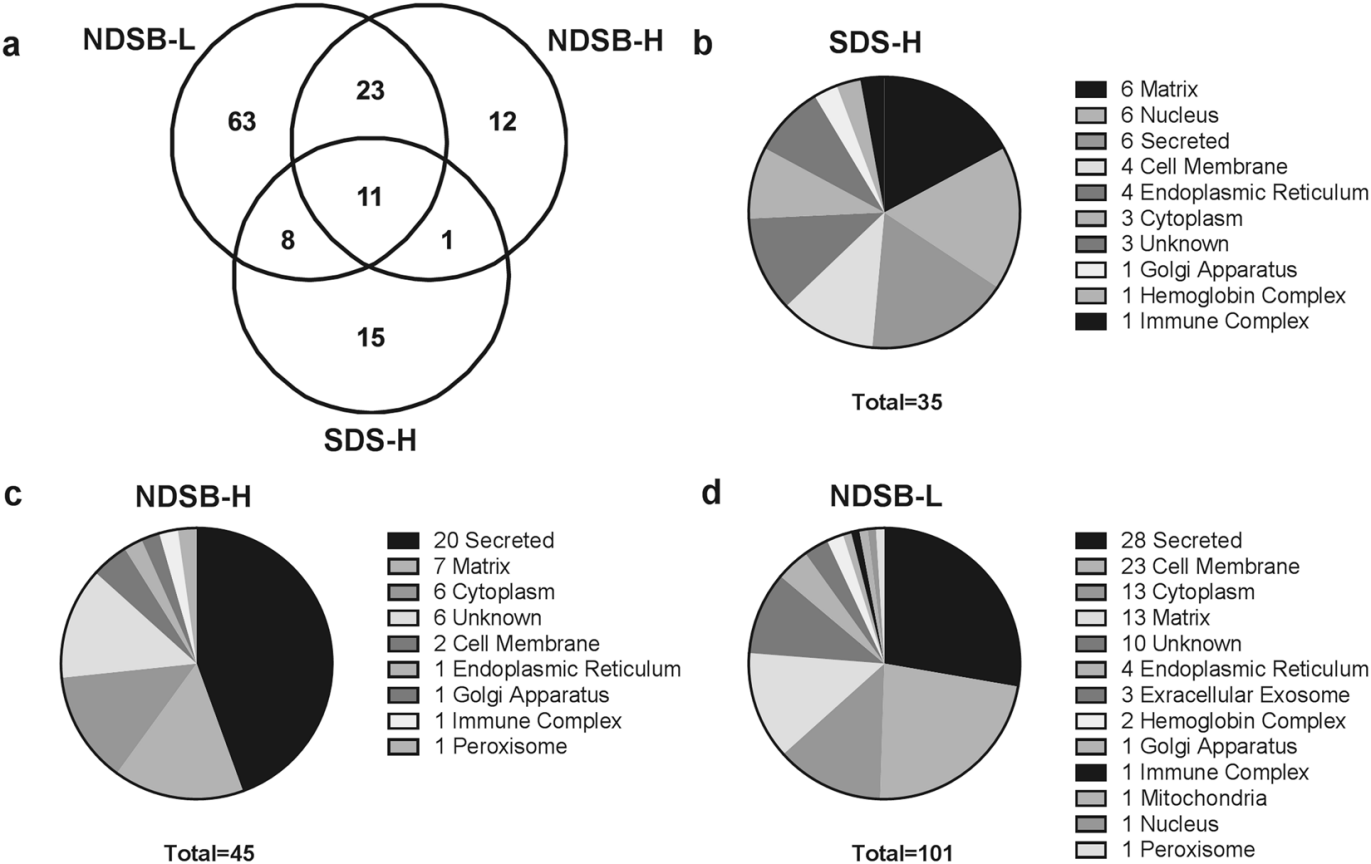
Comparison of antigenic protein identifications depending on type of extraction solution, SDS-H, NDSB-H, and NDSB-L. Venn diagram showing the number of antigenic proteins identified using each protein extraction methods (**a**). Pie charts demonstrating subcellular location of proteins depending on the protein extraction method used (**b**–**d**). Numbers associated with subcellular locations indicate number of antigens identified within that subcellular location. (*n* = 6 per extraction method).

**Figure 5:**
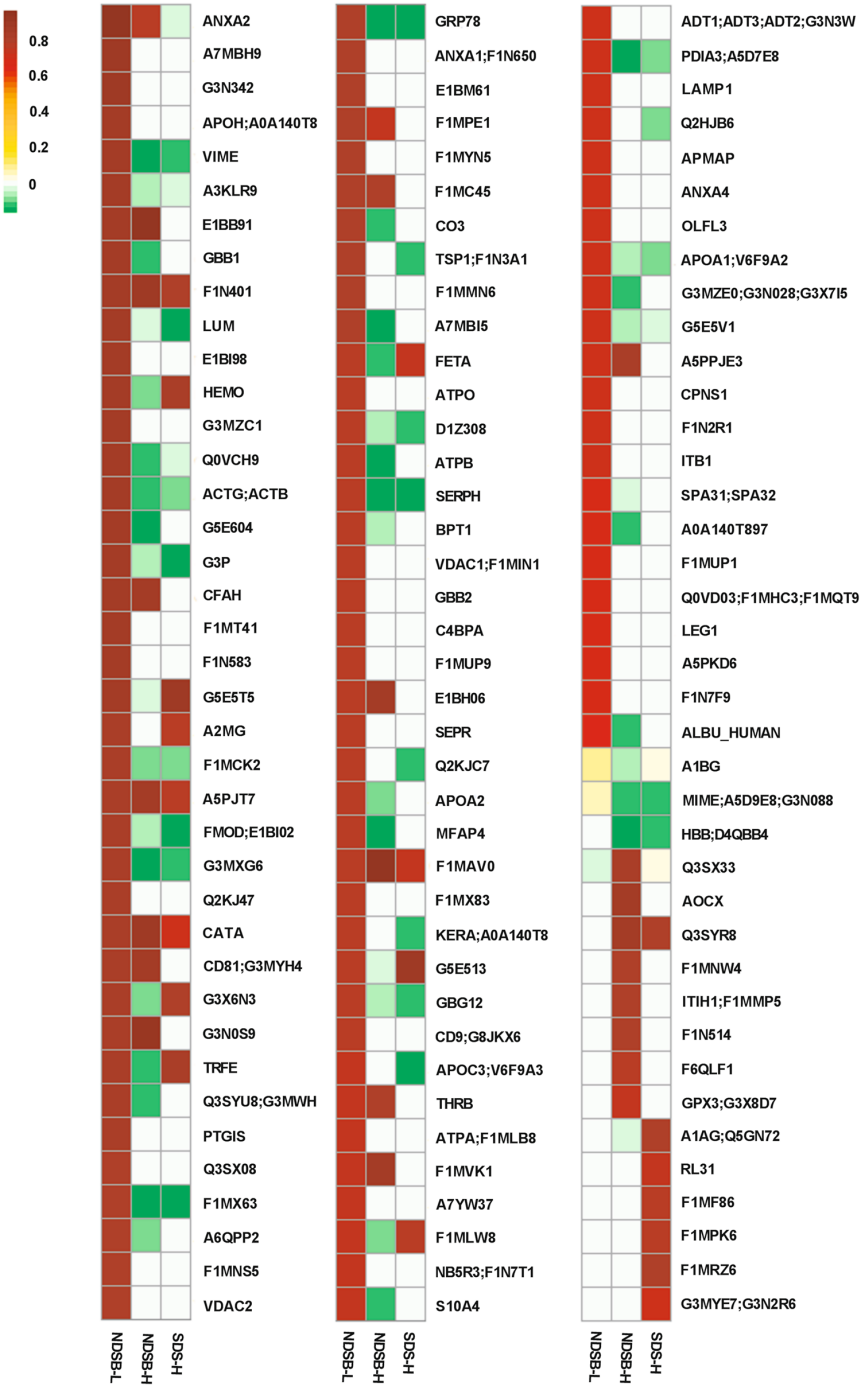
Heatmap of the statistically significant antigenic protein identifications within each extraction method. Antigens were predominantly identified in **NDSB-L**, but alternative extraction methods are required to optimize total number of antigens identified. Scale is a Log of the fold change. Proteomic data was analyzed using Guassian linearized modeling to determine the differential abundance of proteins between the groups as previously reported^60^. White squares indicate that the protein was not present in that sample. (*n* = 6 per extraction method). Groups were considered significantly different when *p* < 0.05.

Mass spectrometry data were analyzed to identify antigenic proteins, and efficiency of antigen identification was examined between protein extraction methods. A total of 133 antigens were identified in native BP (Figs 4 and 5). Despite having the lowest yield of extracted native BP protein (Supplemental Fig. 1), **NDSB-L** protein extracts identified the greatest number of antigens (105), while **NDSB-H** identified 47 and **SDS-H** only 35 (Fig. 4a). Furthermore, **NDSB-L** extracts resulted in identification of 63 unique antigens not present in any other extract. Unique antigen identifications were less commonly found with **NDSB-H** (12 unique identifications) and **SDS-H** (15 unique identifications) extracts. Eleven of the 133 identified antigens were identified using all three extraction methods. Both hydrophilic fractions contained antigens which were also present in **NDSB-L** extracts, whereas only a single identified antigen was found in both **NDSB-H** and **SDS-H** (Immunoglobulin J Chain). Unsurprisingly, more antigens were shared between **NDSB-H** and **NDSB-L**.

Distribution of subcellular locations and biological processes of identified antigenic proteins were investigated using Uniprot database (Figs. 4b–d)^17^. Although antigenic proteins were identified from all tissue compartments and subcellular locations, some locations were overrepresented in terms of their antigen content. When analyzing all 133 antigens, 74.2% were located in the secretions, cell membrane, cytoplasm, and matrix. Importantly, 66% of the total identified cell membrane antigens were integral membrane proteins (18 antigens). Of the remaining antigens, 9% came from the nuclear compartment and endoplasmic reticulum, and 7.6% came from extracellular exosomes, hemoglobin complex, immune complex, golgi apparatus, mitochondria, and peroxisome. Of all 133 antigen identifications, only 9.1% of the total antigens identified were of unknown location.

## Discussion

The present work provides important information characterizing the antigenicity of BP for the use of xenogeneic biomaterials, while establishing and validating a novel affinity chromatography approach for antigen identification. Critically, unlike previously published 2-DE Western blot procedures, we demonstrate that the developed affinity chromatography approach achieves: (1) high-throughput identification of antigenic proteins, (2) compatibility with a wide range of protein solubility’s, most importantly allowing for identification of integral membrane antigens and, (3) increased sensitivity compared to 2-DE immunoproteomic methods. Importantly, we further demonstrate that antigenic components of a commonly utilized xenogeneic biomaterial, BP, are associated with all tissue compartments. These findings carry important implications for the development of unfixed xenogeneic extracellular matrix (ECM) scaffolds as potentially ideal biomaterials for heart valve tissue engineering and regenerative medicine applications^18^. Critically, the finding of non-cellular antigens brings into question the use of tissue acellularity as the primary outcome measure in decellularization approaches^19^. Consequently, the presented findings have application toward the understanding of graft-specific immune responses to xenogeneic biomaterials, for monitoring of such responses in individual patients and for development of new xenogeneic ECM scaffolds in heart valve bioprostheses.

Previously utilized methods for assessment of xenogeneic biomaterial antigenicity have achieved limited success due to their challenging reproducibility and inability to resolve highly lipophilic proteins^13^. Reproducibility of 2-DE approaches and poor specificity of identifications has commonly plagued this technique^20, 21^. In particular, issues such as multiple proteins in a single spot are especially problematic in antigen identification where high specificity is critical^22^. Additionally, highly lipophilic proteins are poorly represented using 2-DE approaches due to their challenging solubility profile particularly during IEF focusing^15^. For example, Beleoken *et al*. found twelve hepatic autoantigens associated with graft versus host disease, however, no integral membrane antigens were identified^23^. Similarly, Biswas *et al*., identified 18 rheumatoid arthritis autoantigens, none of which were integral membrane antigens^24^. Even when utilizing an additional membrane enriching step during protein isolation, Byrne *et al*. found only a single integral membrane antigen out of their 38 putative antigens in a pig-to-primate cardiac transplant model^25^. In previously reported studies of BP, only 9.6% of 31 identified xenoantigens were non-cellular in origin and none were integral membrane antigens^14^. Critically, compared to previously reported 2-DE immunoproteomic methods, the developed affinity chromatography approach was able to identify 18 integral membrane antigens (13.5% of all antigens identified), almost all of which were found in the **NDSB-L** extract (Fig. 5), even though it had the lowest protein yield (Supplemental Fig. 1). Given the difficulty of separating integral membrane proteins on a 2-DE format^13^, not to mention the requirement of additional de-lipidation steps^14^, the affinity chromatography approach utilized here represents an important advance in the field of protein antigen identification. Furthermore, the affinity chromatography approach successfully identified ten BP xenoantigens which had been identified using the previously reported 2-DE immunoproteomic method, including alpha-1-acid glycoprotein, apoplipoprotein A-1, annexin A5, alpha-1B glycoprotein, albumin, beta hemoglobin, hemopexin, superoxide dismutase, mimecan, also known as osteoglycin, and thy-1^14^. It is not surprising that the developed affinity chromatography approach did not identify all of the antigens previously identified using 2-DE immunoproteomic methods, as no proteomic approach is capable of resolving the entire proteome^14, 26^. Excitingly however, more than 100 additional previously unreported BP antigens were identified in this single experiment, indicating greater sensitivity than previous 2-DE methods^14^. The pre-fractionation process was validated by the fact that the majority of the antigens discovered corresponded to their expected subcellular locations. The **NDSB-H** fraction contained predominantly soluble secreted antigens, while **NDSB-L** extracts contained the greatest number of cell membrane associated antigens. Finally, beyond simply overcoming previous immunoproteomic pitfalls, this approach only requires 5–8 hours to run, and has been successfully multiplexed to run up to 28 samples in a single experiment. Therefore, the affinity chromatography antigen identification approach overcomes the limitations of previously published immunoproteomic methods, facilitating rapid, high-throughput identification of both hydrophilic and highly lipophilic (i.e., integral membrane) antigens.

Across antigen identification studies, regardless of disease entity or species under investigation, a relatively small number of antigens are consistently identified, which commonly include isoforms of proteins from the same family^14, 27^. These findings suggest the possibility that shared epitope domains may exist among protein family members. This has been proposed^14^ and validated by other researchers^27, 28^, with one group using epitopes to predict whole antigenic protein families^29^. Indeed, in the present study, since animal models are expected to generate a robust immune response, it is intriguing that a relatively small subset of proteins within BP was identified as antigenic. Furthermore, 31 of the antigens identified in the current study have also been implicated as antigens in the pathogenesis of human diseases^30–35^, the majority of them through identification of their antigen specific antibodies^23–25, 36–49^. A Basic Local Alignment Search Tool (BLAST) search between rabbit and bovine for these previously identified antigens showed them to share between 40–99% homology^50^, with 11 of the antigens sharing greater than 93% homology. This high level of species homology suggests that rather than xenogeneic in-homology determining antigenicity, it is possible that context in which the immune system encounters the antigen is responsible for stimulating the adaptive immune response for many of the identified antigens. Finally, many of the discovered antigens were isoforms of each other such as Annexin A1, A2, and 4; Apolipoprotein A-1, A-11, and C-111; several subunits of ATP synthase; the gamma-12, beta-1 and beta-2 subunits of Guanine nucleotide-binding protein; and Serpin A3–2, Serpin H1 and SERPIND1, which are a part of the Serpin superfamily^51^. Given that so many of the antigens were from the same protein families, a conserved epitope domain could help explain this phenomenon and may represent an interesting target for further investigations. By expanding the number and spectrum of identified antigens, affinity chromatography immunoproteomic methods have the potential to facilitate comparison between antigenic proteins and further fundamental understanding of immune system antigen recognition.

Due to the large number of proteins identified in the current report, a deeper insight into the critical biologic processes associated with graft-specific responses could be gained. Three processes were of particular interest: the immune response, coagulation, and inflammation accounted for 10.5%, 6%, and 3.8% of total antigen identifications. Compare this to the percentage of proteins associated with those processes in the total Bovine UniProt proteome at 4.3%, 0.2%, and 1.5% respectively^17^, it is clear that these processes are overrepresented as antigenic. Furthermore, these processes are not only intertwined but are also involved with the success or failure of biomaterials^52^, and polymorphisms within these systems have been implicated in other disease states^53, 54^. For example, previous investigations have implicated MHC class I polypeptide-related sequence A (MICA), as an immune-related non-human leukocyte antigen (non-HLA)^55^. Future efforts to identify non-HLA antigens may therefore benefit by focusing on immune response, coagulation and inflammation related biological processes. Other processes that were enriched were cell adhesion (6.8% of identifications) and ion transport (5.3% of identifications), compared to UniProt proteome abundance of 1.3% and 0.3% respectively. The importance of these biological processes is currently unknown and therefore warrants future investigation. The finding that specific biologic pathways are overrepresented in antigenic targets has potential to further our understanding of what makes a protein antigenic, which could be vastly beneficial for the field of tissue engineering and control of graft-specific immune responses as a whole.

Currently antigenicity is the major barrier for the use of animal derived tissues in biomaterials. A significant amount of effort has been put into generating screening assays and monitoring tools using galactose-α-1,3-galactose (α-gal)^56^ and other known antigens^57^. But it has been shown that α-gal is not the only source of xenoantigenicity^2^. More success may be found by expanding our knowledge of antigenic tissue constituents. The work here utilized an adjuvanted rabbit model, which allowed for examination of the complete repertoire of potentially immunogenic xenoantigens. Additionally, application of this approach to human patients with glutaraldehyde-fixed and next generation (decellularized/antigen-removed) valves will be required to determine which antigens stimulate graft-specific humoral responses in the unadjuvanted clinical setting. Longitudinal studies may also have value in furthering our understanding of which antigens are primary versus secondary. The current work elucidated important information on preformed IgG antibodies in D0 serum, detected against proteins such as annexin, albumin, and superoxide dismutase. In all cases, antibody response towards these antigens increased longitudinally. Clearly, antigenic proteins are not only of cellular origin but can in fact be intimately associated with the matrix itself, furthering the evidence for a move away from “decellularized” tissues to “antigen-removed” tissues in biomaterial engineering^8, 14^. Increasing understanding of the xenoantigenic determinants of recipient graft-specific immune responses has important implications towards monitoring antibody titers in patients post-implantation, improving development of future xenogeneic biomaterials, and potentially even for development of tolerance induction strategies.

## Methods

All chemicals were purchased from Sigma-Aldrich (St. Louis, MO) unless otherwise stated.

### Tissue Harvest

Fresh BP was harvested immediately postmortem from young adult cattle (Spear Products, Coopersburg, PA, USA) and shipped on dry ice. Tissue was defrosted and washed in 0.1% (w/v) anhydrous ethylenediaminetetraacetic acid (EDTA), 1% (v/v) antibiotic and antimycotic solution (AAS), phosphate-buffered saline (PBS) (pH 7.4) and 4% (v/v) Tris-HCl (pH 8.0). Tissue underwent dissection to remove connective tissue, and the pericardial sac was cut into 1 × 16 cm circumferential strips individually stored in Dulbecco’s Modified Eagles Medium (DMEM) with 15% (v/v) dimethyl sulfoxide (DMSO) at −80 °C^58^.

### Anti-Bovine Pericardium Serum Production

All experimental procedures and protocols were approved by the University of California, Davis Institutional Animal Care and Use Committee (IACUC) and performed in accordance with the relevant guidelines and regulations from the Guide for the Care and Use of Laboratory Animals^59^. New Zealand white rabbits (*n* = 2) received subcutaneous injections of 500 μL of homogenized native BP (1 g BP in 5 mL of 10 mM Tris-HCl (pH 8.0), 1 mM dithiothreitol (DTT), 2 mM magnesium chloride hexahydrate (MgCl_2_ – 6H_2_O), 10 mM potassium chloride (KCl), 0.5 mM Pefabloc (Roche, Indianapolis, IN)) and Freund’s complete adjuvant at a 1:1 ratio at D0, and incomplete adjuvant on D14, 28, 42^58^. Blood was collected on D84, centrifuged at 3000 g, with resultant anti-native BP serum stored at −80 °C.

### Native Bovine Pericardium Protein Extraction

A two-step extraction process was carried out with hydrophilic protein extraction (**H**) followed by lipophilic protein extraction (**L**); using either 134 mM non-detergent sulfobetaine 256 (NDSB-256) (**NDSB-H**) and 1% n-dodecyl-β-D-maltoside (**NDSB-L**), or 0.1% (w/v) sodium dodecyl sulfate (SDS) (**SDS-H**) and 1% (w/v) SDS (**SDS-L**).

Protein was extracted from minced native BP as previously described^58^. Briefly, minced BP (0.2 g) was incubated in 1 mL standard extraction solution (10 mM Tris–HCl (pH 8.0), 1 mM DTT, 2 mM MgCl_2_ – 6H_2_O, 10 mM KCl, 0.5 mM Pefabloc) containing either (1) 134 mM NDSB-256 or (2) 0.1% (w/v) SDS (Bio-Rad, Hercules, CA) representing the two hydrophile extraction solutions. Samples were subjected to 1,000 rpm, 4 °C for 1 h and then centrifuged at 17,000 g, 4 °C for 25 min. Supernatant was collected and defined as **NDSB-H** and **SDS-H** respectively. The insoluble pellet washed twice by resuspension in 1 mL hydrophile extraction solution at 1,400 rpm, 4 °C for 30 min and centrifuged at 17,000 g, 4 °C for 25 min. Supernatant was discarded during wash steps. Washed pellets were then incubated in 0.5 mL lipophile extraction solution containing either (1) 134 mM NDSB-256 and 1% (w/v) n-dodecyl-β-D-maltoside, for **NDSB** pellets or (2) 1% (w/v) SDS for **SDS** pellets. Samples were subjected to 1,400 rpm, 4 °C for 1 h and then centrifuged at 17,000 g, 4 °C for 25 min. Supernatant was collected, defined as **NDSB-L** and **SDS-L** respectively, and stored at −80 °C. Protein concentrations were measured using DC Protein Assay (Bio-rad, Hercules, CA) and protein percent yield was calculated by dividing total protein extracted (mg) by sample weight (mg) and multiplying by 100.

### Affinity Chromatography

IgG antibodies were isolated from whole serum from two individual rabbits using a Protein A HP SpinTrap (GE Healthcare, Pittsburg PA) according to the manufacturer’s antibody capture and cross-linking protocol. The resultant rabbit anti-native BP affinity columns were then utilized to isolate antigenic proteins from native BP protein extracts. Two different types of columns were made, one with D0 serum and one with D84 anti-BP serum. Briefly, serum was diluted 1:10 with binding buffer (50 mM Tris, 150 mM NaCl, pH 7.5), and 200 μL was incubated in the column with end-over-end rotation for 30 min. The column was then washed with 400 μL binding buffer, followed by 400 μL of 200 mM triethanolamine and cross-linked with 400 μL of 50 mM dimethyl pimelimidate dihydrochloride (DMP) in 200 mM triethanolamine for 1 h with end-over-end rotation. Columns were blocked with 400 μL of 100 mM ethanolamine, and unbound antibodies removed with 200 μL of pH 2.9 elution buffer (0.1 M glycine with 2 M urea). Columns were incubated with 200 μL of native BP protein extract for 1 h with end-over-end rotation. Bound proteins were then eluted using sequential washes with elution buffer at a pH of 5 and 4 for non-specific binding and final specific-binding elution at pH of 2.9. All washes and run-throughs were collected for later analysis and stored at −80 °C.

### Serum Electrophoresis

Rabbit IgG antibodies were isolated from whole serum with Protein A HP SpinTrap columns. Native and run-through serum were submitted for serum protein electrophoresis (SPE) through IDEXX (Sacramento, CA) for qualitative assessment of serum IgG depletion (*n* = 1).

### ELISA

Rabbit IgG ELISA Simple Step kits were purchased from Abcam (Cambridge, MA) and used according to manufacturer’s recommendations. Briefly, native serum was diluted 1:1,000,000, while post-capture and post-cross-linking column washes were diluted between 1:1–1:1,000. 50 μL of diluted samples, along with 50 μL of the proprietary Capture and Detection Antibody Cocktail were plated in triplicate in the supplied 96 well plate. Plates were incubated at room temperature (RT) for 1 h, washed 3 times (1,050 μL total) in supplied Wash Solution. 100 μL of Start Solution was added for 3 min and then quenched with 100 μL Stopping Solution. Plate absorbance was read at 450 nm. Column IgG loading capacity was determined as the amount of antibody in native serum minus the amount present in column run-through serum. Column cross-linking efficiency was determined as the percentage of bound antibodies remaining on the column following post-binding and post-cross-linking wash steps. Column binding capacity and cross-linking efficiency were determined using columns for D0 and D84 serum (*n* = 6 per timepoint), and *n* = 3 of technical replicates per wash.

### One-Dimensional Electrophoresis, Western Blot, and Silver Stain

Native BP protein extract, protein run-through and all eluates from D0 and D84 columns were assessed using one-dimensional SDS-page gels and Western blot as previously reported (*n* = 4 per column and protein extraction method)^14, 58^. All blots were probed with D84 serum (1:100 dilution) and assessed for IgG positivity using HRP-conjugated mouse anti-rabbit secondary (1:5,000 dilution) (Jackson ImmunoResearch, West Grove, PA, USA). Additional SDS-page gels were silver stained with a modified acidic silver staining protocol (Silver Stain PlusOne, AmershamPharmacia) (*n* = 4)^14^.

### Proteomic Analysis

Proteins eluted from columns during the specific-binding pH 2.9 elution step were submitted to the University of California, Davis Proteomics Core and subjected to LC-MS/MS analysis. Following proteolytic digestion at a 1:25 ratio of Lys-C/trypsin (Promega) and protein, 550 μL of ammonium bicarbonate was added to dilute urea and activate trypsin overnight at 37 °C. Samples were then subject to liquid chromatography-mass spectrometry on a Exactive Plus Orbitrap Mass Spectrometer (Thermo Scientific) in conjunction with an EASY-nLC II nano UHPLC, a 75 micron, 150 mm silica column filled with Magic C18 200A 3U, and Proxeon nanospray source. Tandem mass spectra were extracted and charge state deconvoluted with IDPicker 2.0, which included a cRAP database of common laboratory contaminants and an equal number of reverse protein sequences (*n* = 6). Distribution of subcellular locations and biological processes of identified antigenic proteins were investigated using Uniprot database^17^.

### Statistical Analysis

Proteomic data was analyzed using Guassian linearized modeling to determine the differential abundance of proteins between the groups as previously reported^60^. Antigenic proteins were defined as those proteins isolated with statistically greater abundance from D84 columns versus D0 columns. A paired, two-tailed Student’s t-test was used to find significance between groups within the ELISA assay. Groups were considered significantly different when *p* < 0.05. Protein extraction yields were analyzed using one-way analysis of variance (ANOVA) with Tukey HSD post-hoc test and statistical significance defined at *p* < 0.05. All values represent the mean ± s.d.

## Electronic supplementary material

Supplementary Information
