# Supplementary material for: Antigenicity of Bovine Pericardium Determined by a Novel Immunoproteomic Approach
Source: Sci Rep. 2017 May 26;7:2446. doi: 10.1038/s41598-017-02719-8 (PMC5446425; doi:10.1038/s41598-017-02719-8)
Supplement: Supplementary file 1 — Supplementary Information [file 41598_2017_2719_MOESM1_ESM.pdf]

Antigenicity of Bovine Pericardium Determined by Novel Immunoproteomic Approach

Katherine V. Gates[a,b *], Ailsa J. Dalgliesh[a,b *], Leigh G. Griffiths[b **]

[a]Department of Veterinary Medicine: Medicine and Epidemiology, University of California, Davis, One Shields Avenue, Davis, CA 95616, USA

[b]Department of Cardiovascular Diseases, Mayo Clinic, 200 First Street SW, Rochester MN 55905, USA

Leigh Griffiths

Department of Cardiovascular Diseases, Mayo Clinic, 200 First Street SW, Stabile 4-58, Rochester MN, 55905. Tel: +1 (507) 774-9732. Fax: +1 (507) 538-6418

E-mail address: Griffiths.Leigh@mayo.edu

* Authors made equal contributions to the study and the publication.

**Corresponding author.

Supplementary Information

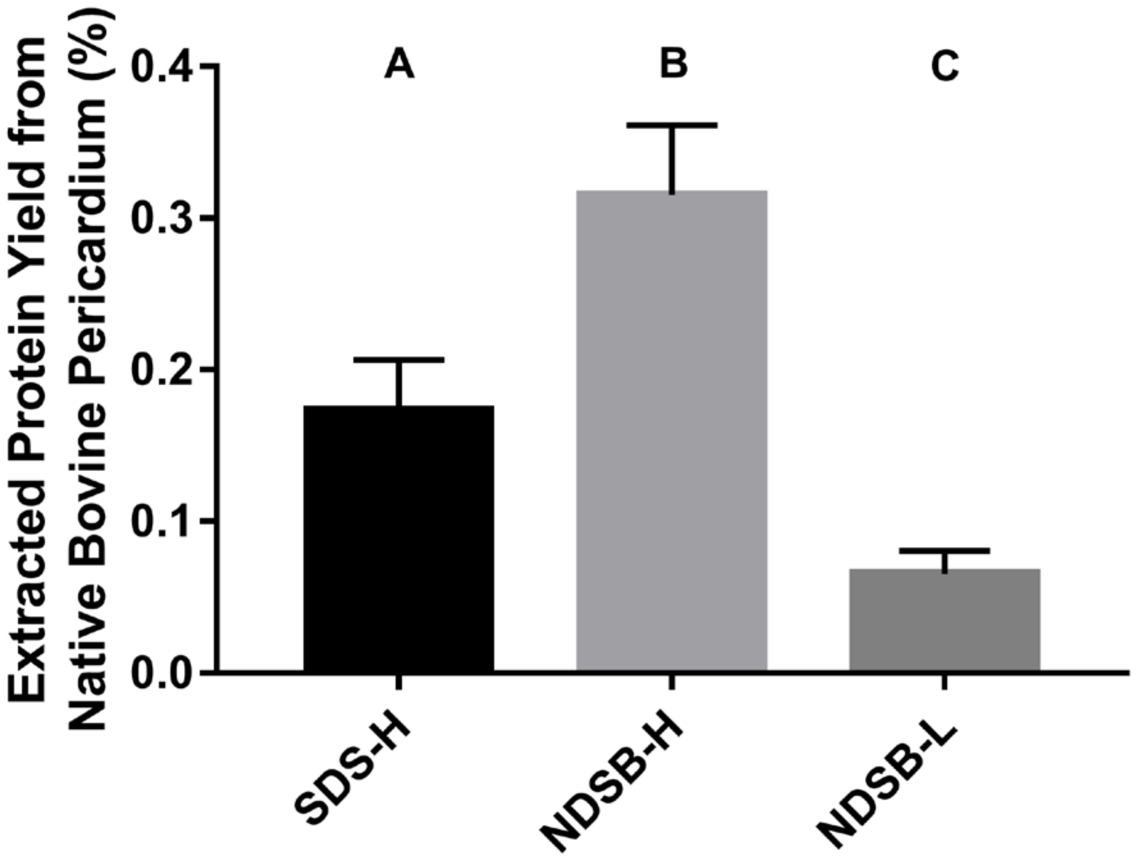

Supplemental Figure 1. Native BP protein extraction % yield achieved using each extraction method. Percent yield of protein extraction for 0.1% (w/v) SDS (**SDS-H**), 134 mM non-detergent sulfobetaine-256 (**NDSB-H**), and 134 mM non-detergent sulfobetaine-256 combined with 1% (w/v) n-dodecyl-β-D-maltoside (**NDSB-L**). **NDSB-L** yields statistically less protein from native BP than either **NDSB-H** or **SDS-H**. All data are expressed as mean ± s.d. and were analyzed using one-way analysis of variance (ANOVA) with Tukey HSD post-hoc test and statistical significance defined at $p < 0.05$ ($n = 8$ for SDS and $n = 10$ for NDSB extraction methods, $p < 0.0001$).

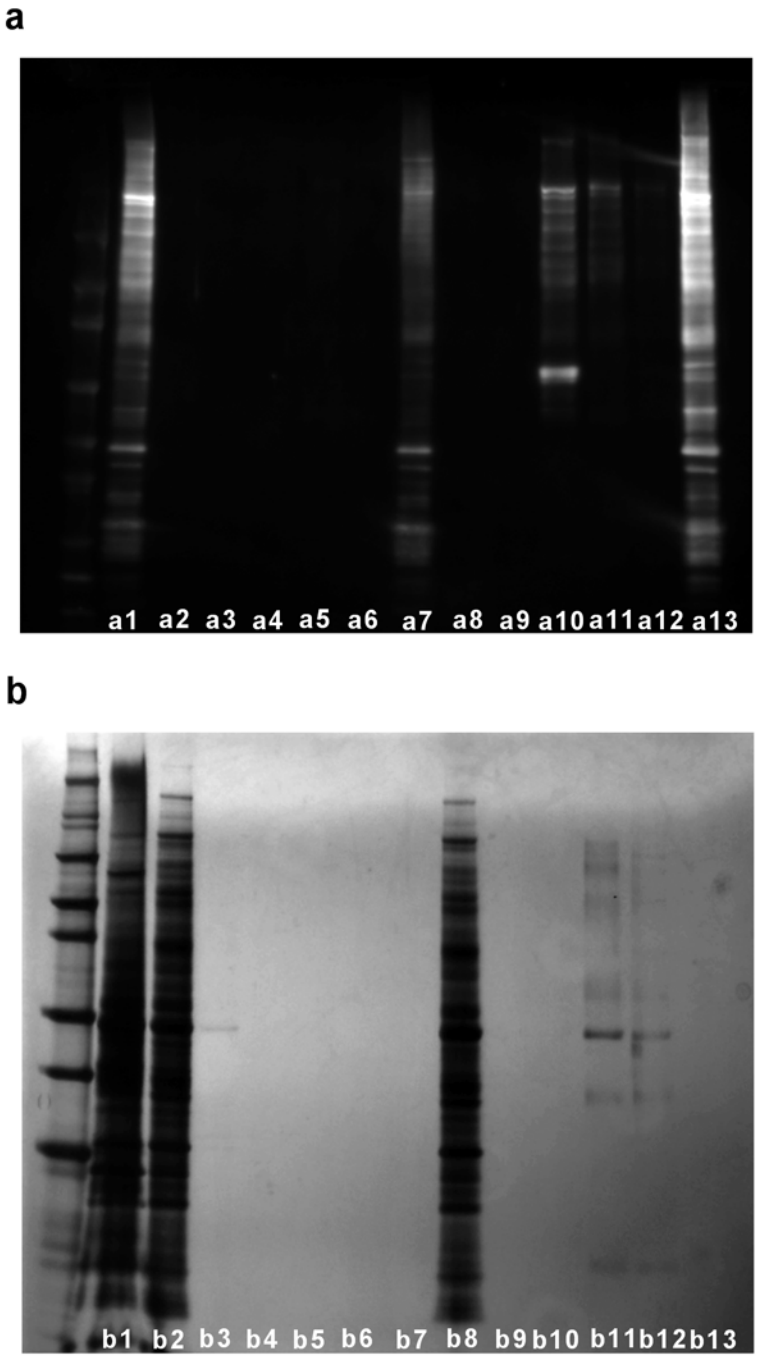

Supplemental Figure 2. Original Western blot and silver stain for the SDS-page gel containing eluent and eluates of Day 0 and Day 84 SpinTrap columns. Western blot (**a**) of total native protein extract (**a13**), protein extract run-through from D0 column (**a1**), and protein

extract run-through from D84 column (**a7**). Both D0 and D84 columns demonstrate decreased antigenic protein content in column run-through, with greater removal of antigenic bands in D84 column. Silver stain (**b**) for SDS-page gel of D84 pH 5, 4, and 2.9 eluates (**b9**, **b10**, and **b11** respectively) and from corresponding D84 Western blot pH 5, 4, and 2.9 eluates (**a8**, **a9**, and **a10** respectively), confirming that minimal non-specific binding is present and that all specifically bound proteins (pH 2.9 eluates) are antigenic. Comparison of D0 and D84 eluates at pH 2.9, demonstrating that more antigens are specifically captured from D84 columns (**a10**) than from D0 columns (**a6**). (*n* = 4).
